# Staggered starts in the race to T cell activation

**DOI:** 10.1016/j.it.2021.09.004

**Published:** 2021-10-11

**Authors:** Arianne C. Richard, Gordon L. Frazer, Claire Y. Ma, Gillian M Griffiths

**Affiliations:** 1 Cambridge Institute for Medical Research, University of Cambridge; 2 Cancer Research UK Cambridge Institute, University of Cambridge

## Abstract

How T lymphocytes tune their responses to different strengths of stimulation is a fundamental question in immunology. Recent work using new optogenetic, single-cell genomic and live-imaging approaches has revealed that stimulation strength controls the rate of individual cell responses within a population. Moreover, these responses have been found to use shared molecular programs, regardless of stimulation strength. However, additional data indicate that stimulation duration or cytokine feedback can impact later gene expression phenotypes of activated cells. In-depth molecular studies have suggested mechanisms by which stimulation strength might modulate the probability of T cell activation. This emerging model allows activating T cells to achieve a wide range of population responses through probabilistic control within individual cells.

## Fine tuning responses with limited components

**Figure 1:**
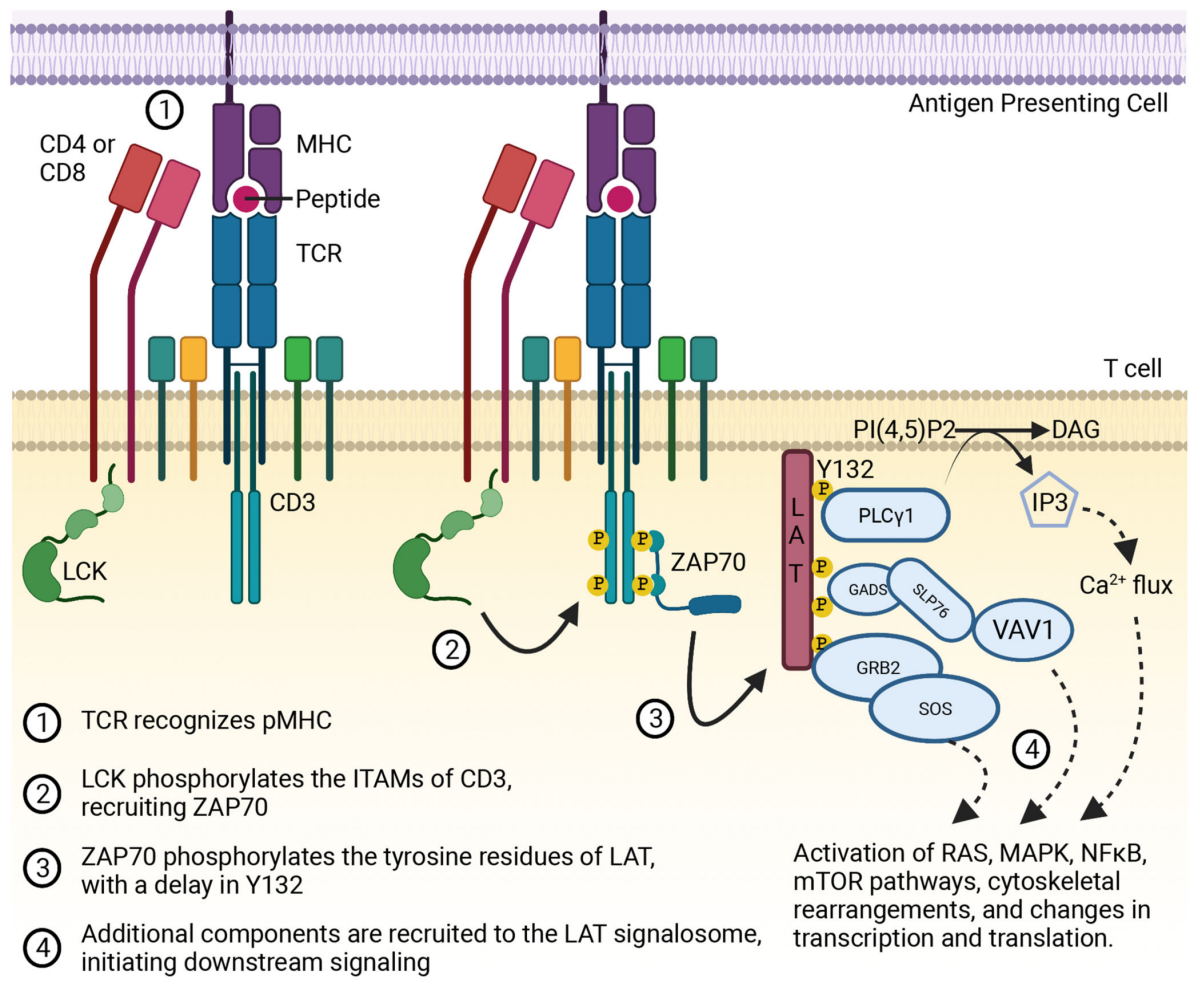
Simplified representation of TCR signaling Cartoon depicts a simplified diagram of initial T cell receptor (TCR) signaling with events listed in temporal order. (1) TCR interaction with pMHC. (2) Recruitment of Lck and phosphorylation of ITAMs of CD3 leading to recruitment of ZAP70. (3) Phosphorylation of the LAT signalosome by ZAP70 and (4) activation of multiple downstream signaling pathways. This figure was made using Biorender.

How T cells meet the challenge of integrating signals from a seemingly infinite array of pathogens with only a limited set of intracellular machinery has long puzzled immunologists. T cell receptors (TCRs) on the cell surface need to sense both the quantity and quality of peptide-MHC (**pMHC**) complexes on antigen presenting cells (APCs), transmitting this information into the cell. TCR ligation rapidly recruits signaling molecules to trigger a broad and interconnected network of signaling events including protein phosphorylation and calcium fluxes^1^ (Figure 1), that initiate a diverse and dynamic range of responses. In **naïve T cells**, antigen recognition stimulates metabolic shifts, transcription, translation, proliferation and differentiation into effector and memory subsets over the course of hours and days; while in **effector T cells**, TCR ligation induces rapid responses (seconds or minutes) including cytokine production and, for **cytotoxic T lymphocytes** (CTLs), secretion of cytolytic proteins at the **immunological synapse** ^2-7^. Each of these **activation events** occurs within individual cells, with the sum of individual responses creating a population response. This review highlights new technologies and the insights they have revealed, suggesting how TCR-pMHC interactions in single cells can generate finely-tuned activation responses within a population, and focusing on the early hours after TCR ligation in naïve and effector T cells.

## Manipulation of T cell stimulation strength

The stimulation strength that an individual T cell senses can be impacted by both the concentration of pMHC ligands, as well as their affinity for the TCR^8^. One of the earliest examples of altered **stimulation strength** came from characterization of the TCR agonist antibody OKT3, which demonstrated concentration-dependent effects on human T cell proliferation^9^. Sensitivity of T cell responses to single amino acid changes in the peptide ligand was first established in experiments stimulating polyclonal T cell populations from inbred mice^10^. TCR gene cloning then allowed a more detailed investigation of the binding properties and biological effects of subtly altered ligands^11-16^. Although stimulation strength generally correlates with ligand affinity, observations of high-affinity yet low-potency ligands alongside single-molecule force measurements have led to the proposal that potency is actually determined by the formation of catch- versus slip-bonds between TCRs and pMHC ligands^17^, but the existence of these different structures continues to be debated ^18-22^. The resolution achievable using TCR-transgenic systems is inherently limited by the ability to find an **altered peptide ligand** (APL) that exhibits the desired binding behavior, making questions about specific lengths of pMHC engagement or patterns of binding and re-binding events difficult to answer. To circumvent this issue, several groups have recently developed **optogenetic** receptor-ligand systems in which binding kinetics are controlled by light patterns^23-26^ (Box 1). While the synthetic nature of optogenetic systems must be considered in interpreting results, these methods enable a new level of precision in dissecting the temporal binding requirements of T cell activation.

In addition to signaling through the TCR, inputs from a multitude of costimulatory and cytokine receptors can modulate the strength of stimulation that a T cell experiences. Ligation of costimulatory receptors including CD28, CD27, and CD2 can augment TCR signals and enhance activation^27-30^. These effects may be particularly important for cells receiving weak TCR signals, as exemplified by CD27 ligation enhancing proliferation in murine CD8^+^ T cells stimulated by reduced affinity TCR ligands^27^. Likewise, cytokine signaling can synergize with TCR-induced signals^31-34^. The ways in which these additional stimuli impact T cell responses are diverse. For example, TCR and costimulatory/cytokine signaling showed additive effects on proliferation potential in experiments using division tracking dyes during activation of naïve murine CD8^+^ T cells^29,35^. In contrast, costimulatory receptor engagement rescued cytokine expression in primary human CD8^+^ T cells under chronic *in vitro* stimulation^36^. Much remains to be understood about how costimulatory and cytokine signals integrate into T cell activation signaling to control the effective stimulation strength that a T cell experiences. One highly studied example is the cytokine IL-2, which is expressed in a stimulation-strength-dependent manner by both CD4^+^ and CD8^+^ T cells and results in both autocrine and paracrine signaling through IL-2R^34,37,38^. Experiments in naïve murine CD8^+^ T cells demonstrated that adding exogenous IL-2 can rescue translation and proliferation deficiencies seen in cells stimulated with low dose or low affinity ligands^31,32^. This is likely achieved by promoting the expression of the transcription factor MYC, which requires ongoing protein synthesis due to rapid turn-over and controls division potential^31,32,39,40^. Future work examining the integration of other signals can thus shed light on the regulatory logic of intracellular T cell signaling.

A plethora of studies have demonstrated that reducing stimulation strength during activation of naïve or effector CD4^+^ or CD8^+^ T cells leads to a reduction in activation phenotypes including signaling protein phosphorylation, calcium fluxes, transcription factor activation, mRNA expression, protein expression, proliferation, cytokine secretion and cytolytic activity, as exemplified by refs^41-50^. The strength of T cell stimulation can also dramatically impact thymic selection, which falls outside the scope of this review^51^.

## Insights from early single-cell measurements

Historically, RNA and protein expression measurements were made on bulk cellular lysates, and functional tests used pools of T cells. These types of measurements describe the average behavior of a population but cannot discern how individual cells are affected. Thus, a reduction in average cellular activation in a given condition might be due to a change in the magnitude of activation within each cell, or to a change in the proportion of cells that are activated. Single-cell measurements are able to overcome this issue and provide more accurate insights into how individual cell responses combine to achieve a population response.

One of the original single-cell methods, **flow cytometry**, enables quantitative read-outs of protein expression or modification in individual cells using fluorescently-tagged antibodies, constructs or dyes. This approach has revealed that some markers of activation exhibit simple “on/off” behavior, such that the proportion of “on” cells changes with stimulation strength. This type of response, termed “**digital**”, is exemplified in primary murine T cell activation by the phosphorylation of kinases such as extracellular signal-related kinase (ERK)^52,53^ and protein kinase D2 (PKD2)^54^. Other markers of activation show a graded response such that increasing stimulation strength shifts the marker intensity within each individual cell. IRF4 expression is the best characterized of these “**analog**” responses, with extensive studies in murine CD8^+^ T cells^55-58^. Recently, a hybrid digital/analog model has been used to describe certain activation markers that exhibit both “on/off” behavior and graded modulation of intensity within the “on” population (e.g. expression of CD69 in CD4^+^ T cells^59^ and MYC in CD8^+^ T cells^39,40^). For these markers, both the percentage of positive cells and the intensity of the positive population are influenced by stimulation strength^39,40,59^. The existence of such hybrid behaviors suggests that the digital/analog dichotomy may be overly simplistic. This is particularly relevant for gene expression changes, which can accumulate over the course of active signaling^23^, as described in detail below.

While flow cytometry has been instrumental in revealing these activation behaviors, its early use had two major drawbacks. First, early flow cytometry methods produced uni- or oligo-dimensional measurements, leaving the relationships between activation events within individual cells unclear. (The number of measurable parameters has gradually increased over time and has recently been expanded even further through spectral flow cytometry, as described in Box 2). Second, measurements are static, making it impossible to know whether cells are in transition or steady-state. For example, increased prevalence of an intermediate phenotype among weakly stimulated cells might indicate a stable state of partial activation or might reflect a reduced speed of response. Likewise, altered proportions of activated cells might indicate a change in steady-state proportions or might be caused by a shift in the **activation rate** (events per unit time) of a response. We argue that these distinctions are crucial when testing the impact of stimulation strength on activation phenotypes, as they can lead to different interpretations of how the underlying intracellular machinery reads TCR signals.

## Advances in single-cell measurements reveal a rate-based model of T cell activation

**Figure 2:**
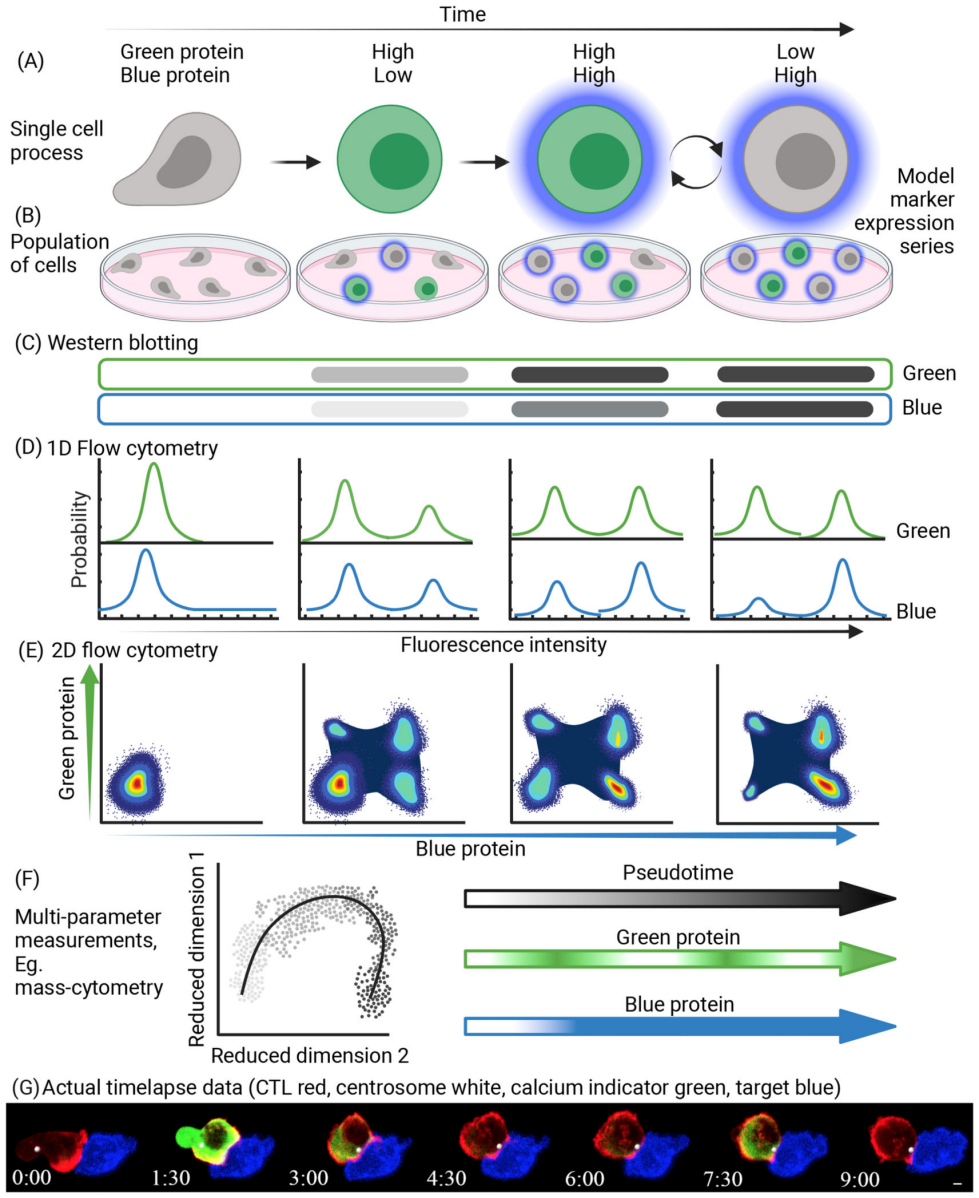
Cell population approaches versus single cell approaches over time (A) A schematic representation of a theoretical T cell activation series of events. In this model, during activation the T cell first upregulates Green Protein, then Blue Protein. The expression of Green Protein then oscillates (round arrows) between low and high expression. (B) Model of how expression of these proteins might look by fixed cell imaging. When T cells are activated in a population, all of the states in (A) may be represented and vary with time. (C) Model of how expression of these proteins (Green, top; Blue, bottom) might look by Western blot of pooled cell lysates. (D) Model of how expression of these proteins (Green, top; Blue, bottom) in the population might look by single parameter flow cytometry. (E) Model of how expression of these proteins (Green, y-axis; Blue, x-axis) in the population might look by multi-parameter flow cytometry. (F) Model of how expression of these proteins (Green, right middle; Blue, right bottom) might look following a high dimensional data capture technique such as mass cytometry or single cell RNA sequencing (scRNA-seq) with simultaneous protein measurements. Note that the extra parameters allow inference of a pseudotime trajectory (left and right top) that reveals the different expression dynamics of Blue and Green proteins. However, as it is a pseudotime trajectory constructed from snap-shot measurements, it cannot elucidate precise timescales of expression. Only through continuous time-lapse imaging is the full activation behavior readily apparent. (G) To illustrate the benefits of live imaging in individual cells, we show a time-lapse series of a cytotoxic T lymphocyte (CTL) (red) interacting with an antigen-presenting target cell (blue) captured with a spinning disk confocal microscope. As the CTL interacts with the target cell, a calcium flux is initiated, shown by the oscillating green intensity proportional to the free intracellular calcium, and the centrosome (white sphere) polarizes toward the immune synapse. While calcium may also be measured by alternative approaches, oscillatory behavior in an individual cell requires live imaging. Moreover, organelle movement such as polarization of the centrosome can only be measured through visualization. Scale bar=2μm, Time Min:Sec. This figure was made using Biorender.

The advent of high-dimensional single-cell technologies including single-cell RNA sequencing (scRNA-seq) and mass cytometry (Box 2), as well as advances in live cell imaging (Box 3), have facilitated more comprehensive profiling of T cell activation to uncover the dynamics of individual cell responses (Figure 2).

In naïve T cells, one of the primary outcomes of TCR stimulation is the induction of gene expression. A recent study scRNA-seq study examined the transcriptional changes downstream of *in vitro* naïve CD8^+^ T cell activation^3^, using the OTI TCR-transgenic mouse system^12^, in which all T cells are specific for an ovalbumin peptide and for which APLs of varied affinities have been well-characterized^60^. Using **pseudotime** analyses to compare the activation progress of cells stimulated with different APLs, this study demonstrated that transcriptional responses to strong stimulation were rapid and synchronized while responses to weak stimulation were more temporally heterogeneous and on average delayed^3^. However, the transcriptional activation trajectory was largely shared, regardless of stimulation strength, suggesting that this process is utilized by all activating cells. These results indicate that stimulation strength can impact the rate with which cells initiate transcriptional activation.

A subsequent mass cytometry study looked upstream of transcriptional activation and asked how signaling events marked by protein phosphorylation and degradation across multiple T cell signaling pathways were influenced by ligand affinity in the same OTI T cell activation system^61^. This approach revealed a set of signaling events that were shared among cells regardless of stimulation strength, but which were, on average, delayed with weaker stimuli. These results echo the transcriptional findings that stimulation strength can control the rate with which cells initiate a shared activation program. Due to the rapidity and transience of proximal signaling events, this latter study focused on TCR-distal signaling nodes, honing in on the coordination of ERK, S6 and STAT5 phosphorylation^61^, and future work examining simultaneous activation of TCR-proximal signaling mediators will be important to understand the initiation of this shared downstream signaling program.

Similar conservation of T cell activation processes was observed in flow cytometry experiments examining markers of metabolic shift and cell cycle entry in OTI TCR-transgenic cells stimulated with APLs of varied affinities^62^. Likewise, results from these high-dimensional single-cell studies are reminiscent of earlier work that used division-tracking dye to monitor proliferation of APL-stimulated OTI T cells and found that the rate of proliferation entry, but not the speed of ongoing proliferation, was dependent on stimulation strength^50^. Together these studies demonstrate that under controlled *in vitro* settings, stimulation strength can regulate the rate of activation in naïve CD8^+^ T cells. Comparison with *in vivo* studies will be important to understand how such a mechanism plays out in a complex physiological environment.

**Figure 3:**
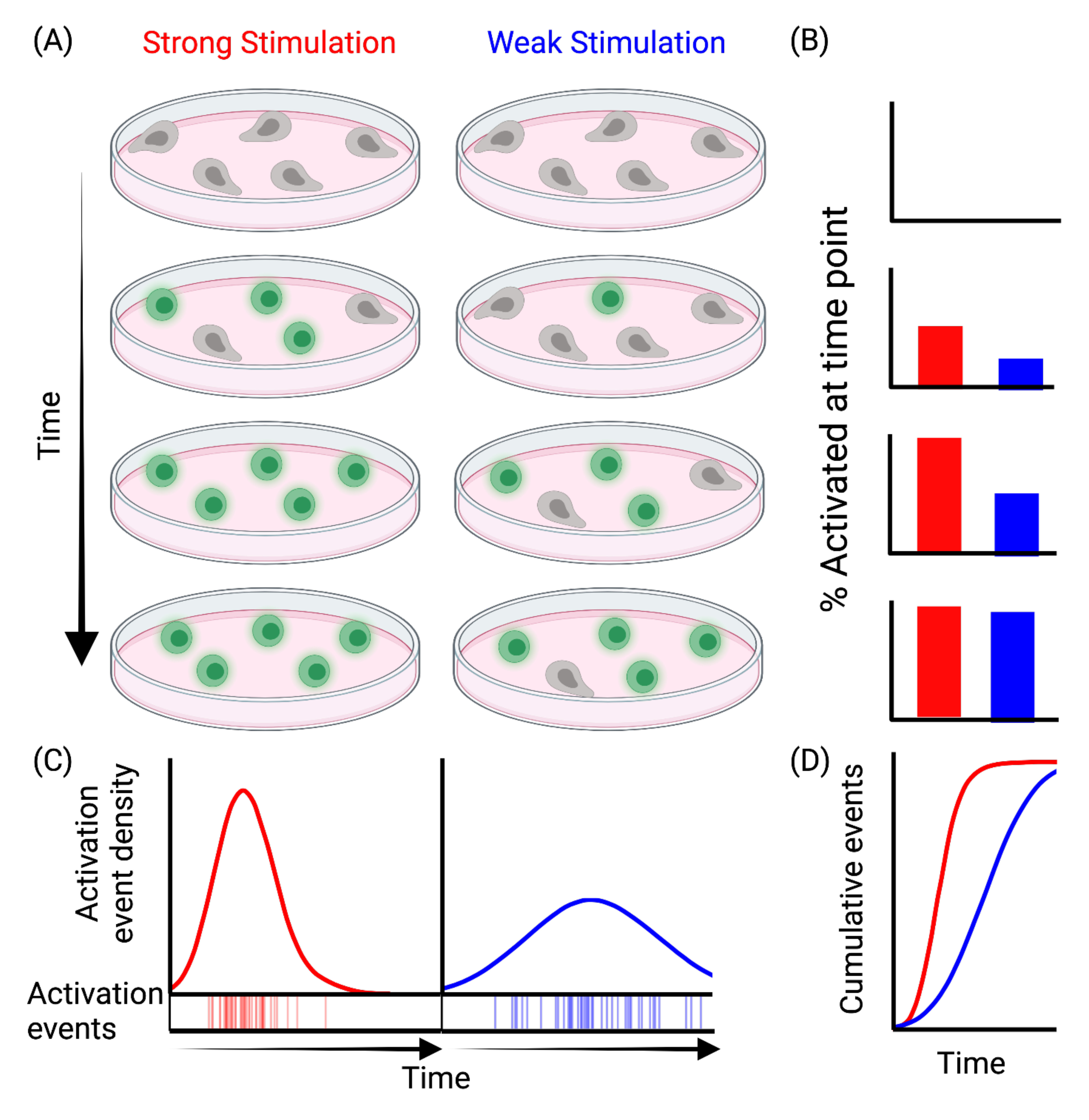
Model of a rate-based mechanism of T cell activation (A) A schematic representation of a theoretical T cell activation series in populations responding to Strong (red) or Weak (blue) stimulation. (B) Bar chart representation of how the percentage of activated cells at each timepoint might look comparing Strong (red, left) v Weak (blue, right) stimulation. (C) Simulated model of T cell activation with Strong (red, left) v Weak (blue, right) stimulation where stimulation strength controls the rate of activation events. (D) Cumulative distribution curves for simulated data from (C). This figure was made using Biorender.

In effector CTLs, TCR ligation initiates cytokine secretion and targeted killing of the antigen-spresenting cell. In order to kill a target cell, a CTL undergoes substantial cytoskeletal reorganization to polarize its centrosome toward the target and deliver cytolytic granules to the immunological synapse^6^. One study used confocal live imaging of *in vitro-*activated OTI TCR-transgenic CTLs to monitor the impact of ligand affinity on the dynamics of the CTL-target cell interaction and the intracellular movement of the centrosome and granules^63^. Data showed that TCR stimulation strength was associated with the proportion of cells exhibiting long **dwell times**, sustained calcium fluxes, docked centrosomes, and polarized granules. However, within cells that achieved long dwell times and organelle polarization, the organization and speed of the response was independent of stimulation strength, suggesting a conserved activation program. These results suggest that, as in naïve T cells, stimulation strength controls the rate of effector CTL activation. Such a rate-based model (Figure 3) might help explain the fact that even extremely weak TCR stimulation can induce rare occurrences of activation in naïve, memory and *in vitro-*activated T cells from both humans and mice^16^. It will be interesting to see whether studies in other systems conform to this rate-based model.

The picture emerging from the single-cell studies described above is that T cells utilize remarkably fixed intracellular activation programs (Figure 3). Supporting this conclusion, recent experiments using recombinant pMHC ligands to stimulate primary human CD8^+^ T cell blasts expressing an exogenous TCR showed that the antigen dose threshold for the production of multiple cytokines was always shared, regardless of ligand affinity^28^. The authors of this study further validated their findings in primary human memory T cells stimulated with peptide-pulsed monocyte-derived dendritic cells^28^. However, earlier studies that varied ligand affinity and dose during stimulation of *in vitro*-maintained human and mouse CD8^+^ and CD4^+^ T cell clones observed dose-response hierarchies instead of a shared stimulation strength threshold among cytokines^64-66^. The reason for this discrepancy is unclear but may reflect differences in the experimental systems used.

Additional evidence of stimulus-dependent tuning beyond a shared activation program comes from studies of TCR-induced gene expression changes. In the scRNA-seq study described above, after accounting for each cell’s activation status, a small number of genes remained differentially expressed at the mRNA level between cells stimulated by strong and weaker ligands^3^. Likewise, observations of hybrid digital/analog expression of induced proteins support the idea of tuning beyond a shared response, such that a common program initiates expression in a digital manner and subsequent stimulus-dependent effects tune this expression in an analog manner within each cell (e.g. refs^40,59^). Moreover, extensive work using APLs to stimulate naïve CD8^+^ T cells in multiple murine TCR-transgenic systems has shown that starting from approximately one day after activation, T cells express IRF4 in a graded manner reflecting stimulation strength^55-58^. As IRF4 can enhance effector differentiation^57,58^, this suggests that subtle tuning of gene expression in the early days of naïve T cell activation might alter differentiation outcomes, as has been observed in *in vivo* models for both CD4^+^ and CD8^+^ T cells^47,67-72^. Similarly, experiments stimulating murine CD4^+^ T cells with varying antigen doses found that after 24 hours, stimulation strength correlated with the expression of IL-12Rß2, which facilitates Th1 polarization in response to IL-12 signaling^72^. These results again suggest a means by which stimulation strength can impact differentiation fate. Together, these data indicate that although shared activation programs may exist, the strength of T cell stimulation can further tune resulting activated T cell phenotypes.

This raises the important question, if the rate-based model for activation is accurate, how are responses tuned beyond a core activation program according to stimulus? One potential explanation is that cells continue to receive stimulation beyond an initial activation event. An elegant optogenetic study tested the impact of sustained signaling on T cell activation responses^23^. Using an optogenetic **chimeric antigen receptor** (optoCAR), in which light induced the dissociation of the intracellular signaling moiety from the receptor-ligand complex and its subsequent inactivation, the authors quantified the persistence of TCR-induced signals including calcium flux, ERK and FOS phosphorylation, and gene transcription, in the human Jurkat T cell line. Results showed that upon proximal signaling disruption, downstream activation events rapidly dissipated, but sustained signaling led to the accumulation of gene expression outputs in the hours following activation. While studies in primary cells using TCRs will be required to determine the generalizability of these findings, they suggest that stimulation strength could impact mRNA and protein expression phenotypes by altering the effective duration of stimulation that cells experience.

An alternative though not mutually exclusive explanation for stimulation strength-dependent response tuning is that the T cell microenvironment, and thus the additional signals the cell receives, changes with stimulation strength. For example, previous work combining *in vitro* stimulation of murine TCR-transgenic CD4^+^ T cells with mathematical modeling found that the availability of the effector-promoting cytokine IL-2^73^ is carefully regulated according to antigen dose through multiple feedback loops^38^. (Indeed, for this very reason, many studies aiming to explore cell-intrinsic effects of stimulation strength attempt to overcome IL-2 feedback, e.g. refs^3,50,61^.) Such differences in the cytokine milieu might mediate strength-dependent cellular responses, particularly at later time points when stimulation-induced cytokines could feed back on the activating cells. Further exploration of tuning behaviors and the conditions in which they are observed will thus be important to better understand the full impacts of stimulation strength on T cell activation.

## Stimulation strength can control the probability of “turning-on” T cell activation

Observations that stimulation strength can control the rate with which T cells initiate a core activation program suggest a switch-like mechanism at some stage of the TCR-induced signaling pathway where the decision to signal further downstream is made. Recent live-imaging work has shed light on the TCR ligation properties that modulate this switch. Specifically, one study used TIRF microscopy to image individual TCR-pMHC interactions between murine TCR-transgenic CD4^+^ T cells specific for a peptide from moth cytochrome C and pMHC on **supported planar lipid bilayers** additionally functionalized with ICAM-1^74^. They found a wide distribution of receptor-ligand dwell times, the mean of which corresponded to TCR-pMHC affinity. Moreover, measurements of nuclear translocation of the transcription factor NFAT (as an activation marker) revealed that successful activation was associated with either a single long dwell time, or sequential, short, spatially-correlated binding events. In this way, all activated cells received the same total input, regardless of ligand affinity. The model suggested by this study is that activation events occur in a probabilistic manner, taking place when sufficiently long real or effective dwell times are stochastically achieved. This interpretation provides an intriguing mechanism that might explain how ligand affinity as well as concentration can alter the rate of cellular activation.

Theoretically, converting TCR-pMHC binding dwell times into a highly discriminatory activation switch requires a thresholding mechanism. One of the most popular models for this is **kinetic proofreading**, which posits that signaling steps introduce a delay between ligand binding and subsequent activation cascades, such that weak interactions often dissociate before responses are triggered^75-77^. Two recent optogenetic studies explicitly tested the concept of kinetic proofreading in T cells using light to alter the binding half-lives of synthetic ligand-receptor pairs in an otherwise uniform environment^25,26^. One study used a **LOVTRAP system** in which a CAR expressed in Jurkat cells was bound in a light-controlled manner to LOV2 presented on a supported lipid bilayer^25^. The second study used a **PhyB/PIF system** in which Jurkat cells expressed a construct of PIF6 fused to a TCRß chain that underwent light-controlled binding to PhyB tetramers^26^. Both studies found that longer binding half-lives resulted in greater activation, even when controlling for receptor occupancy^25,26^, consistent with the kinetic proofreading model. However, it must be noted that there are many differences between these synthetic receptor systems and native T cell-APC interactions, which include coreceptors and adhesion molecules among other factors. Thus, continued testing of the model in native systems is merited.

The stage in the signaling network at which kinetic proofreading might be achieved also remains unclear. A recent study combined *in vitro* experiments and mathematical modeling to calculate the number of steps required for kinetic proofreading^16^. The authors varied ligand dose and affinity while stimulating primary human CD8^+^ T cells expressing an exogenous TCR, and then fitted a model of a kinetic proofreading mechanism. This yielded an estimate that the delay from initial TCR binding to activation takes 2.8 seconds and 2.67 biochemical steps (the fractional number may reflect delayed reversion of one or more steps upon ligand dissociation). These results suggest that if a molecular switch exists, it occurs early in the signaling pathway. Testing whether data from other T cell stimulation systems yield the same parameter estimates will be important to gauge the generalizability of this conclusion.

Following TCR ligation, phosphorylation of immunoreceptor tyrosine-based activation motifs (ITAMs) in the intracellular portions of CD3 subunits initiates signaling cascades^78^. Experiments varying the number of ITAMs on synthetic receptors expressed in Jurkat cells found that increasing the number of ITAMs increased the proportion of cells exhibiting activation phenotypes, including NFAT reporter expression and ERK phosphorylation, and the synchronicity of activation^79^. These results appear similar to those seen with increasing ligand affinity, suggesting that the signal strength conferred by ligand affinity might impact the efficiency of TCR ITAM phosphorylation. Following ITAM phosphorylation, ZAP70 is recruited and activated^78^. Experiments using the LOVTRAP optoCAR described above and measuring ZAP70 recruitment and diacylglycerol (DAG) accumulation in response to varied dwell times and receptor occupancy levels revealed no evidence of kinetic proofreading at the level of ZAP70 recruitment, but offered strong evidence of this mechanism further downstream at the level of DAG accumulation^25^. These data suggest that the putative molecular switch is between these two activation events in this optoCAR system and provide a hypothesis for testing in intact T cells.

Downstream of ZAP70, the **LAT signalosome** assembles, recruiting and activating multiple signaling intermediates including Phospholipase C gamma 1 (PLCγl), which cleaves phosphatidylinositol 4,5-bisphosphate (PI(4,5)P2) to generate DAG and inositol 1,4,5-trisphosphate (IP3). Intriguing recent evidence suggests that phosphorylation of LAT Y132 in humans (LAT Y136 in mice) might be responsible for initiating the T cell activation program^80^ (reviewed in ref^81^). Phosphorylation of most LAT tyrosines is promoted by neighboring acidic residues^82^, but Y132 is an exception and is phosphorylated at a slower rate^80,83^. The substitution of an acidic residue next to Y132 was sufficient to enhance its phosphorylation rate, and increase the phosphorylation, recruitment, and activation of PLCγ1 ^80,82^. With this modification, both Jurkat cells and primary murine CD8^+^ T cells that had no or poor responses to weak ligand stimulation in the absence of the acidic residue now activated^80^. These results suggest that this LAT phosphorylation event might act as a molecular switch controlling T cell activation. A subsequent mathematical modeling study confirmed the importance of this phosphorylation step in ligand discrimination and hypothesized numerically-supported mechanisms by which it might either form a kinetic proofreading step itself, or sustain proofreading from an earlier step^84^. This study also highlighted the necessity of spatial colocalization of proximal signaling mediators to achieve kinetic proofreading. Together these results raise the possibility that kinetic proofreading might occur at or upstream of the slow phosphorylation of LAT Y132, focusing the field for future investigations.

Further evidence that PLCγ1 recruitment to the LAT signalosome may mark a turning point in T cell activation comes from both its interaction with LAT and its ability to cleave PI(4,5)P2 into DAG and IP3, which drives the calcium flux. First, experiments monitoring the condensation of LAT on supported lipid bilayers in the presence of GRB2 and SOS showed that the formation of LAT aggregates can act as a rate-limiting step in the activation of RAS^85^, an event that was further exacerbated by the addition of PLCγ1^86^. Second, a confocal live imaging study in murine *in vitro-*differentiated CTLs found that the catalytic activity of PLCγ1 at the CTL immune synapse drives a positive feedback mechanism^87^. This study tracked lipid modifications at the immunological synapse over time and exogenously expressed a modified PIP5K with constitutive synapse localization. Their results showed that the PLCγ1-induced reduction in negatively charged PI(4,5)P2 causes a loss of electrostatically bound PIP5K, preventing regeneration of the negative charge and depleting the actin mesh across the synapse, allowing granule secretion to occur. Subsequent work in the same system showed that reducing stimulation strength reduced the area of synapse depleted of negative charge and actin, as well as the proportion of cells capable of achieving this depletion^63^, suggesting that the efficiency of this process depends on stimulation strength. Finally, though simultaneous imaging of centrosome movement and calcium flux, this latter study revealed a calcium flux threshold associated with centrosome docking^63^, which, together with previous reports implicating both DAG and the calcium flux in centrosome polarization^88-94^, suggests a tipping point at or upstream of PLCγ1. Thus, although it remains an open question, there is increasing evidence to suggest that slow modification of LAT and recruitment of PLCγ1 might constitute a gateway to downstream activation programs, converting stimulation strength into a probability of activation at the single-cell level.

## Concluding Remarks

Recent advances in single-cell genomic and imaging technologies, combined with greater control over T cell stimulation, have enabled researchers to revisit T cell activation questions from a newly dynamic and granular perspective. This vantagepoint has revealed that stimulation strength can impact the rate with which cells utilize a common set of activation programs. These observations can reconcile results from previous studies using bulk, static, or uni-dimensional measurements that found differences in the speed, magnitude, or proportion of T cell responses. Such a mechanism is intellectually appealing as it enables a wide range of T cell responses at the population level without requiring infinitely diverse responses from each individual cell. A **probabilistic model** for initiating a molecular program has been proposed in the context of *in vivo* T cell differentiation, where individual cells exhibited extensive heterogeneity in their progress along a shared differentiation trajectory, but the combined population response was highly robust^95^. Moreover, the use of fixed molecular programs has precedent in other biological systems, for example development, where integrated signaling networks can control activation of a consistent set of differentiation pathways^96,97^. As T cells continue to integrate signals from their TCR and other environment-sensing receptors, it is highly likely that further tuning of responses takes place beyond a core activation program – the mechanisms of which are not fully understood. It also remains unclear how tunable activation responses are at the individual cell level. As described in the **Outstanding Questions**, further dissection through use of these and other emerging technologies will continue to shed light on the regulatory logic governing T cell responses, which may benefit our understanding of diseases driven by inappropriate T cell activation as well as inform the rational design of T cell-targeting therapeutics or vaccines.

## Highlights

- Advances in optogenetic models, single-cell genomics, and live imaging allow new levels of precision in testing the impact of stimulation strength on T cell activation.
- Single cell studies have revealed that stimulation strength modulates the rate at which both naïve and effector T cells initiate fixed activation programs.
- Optogenetic experiments have shed light on the receptor–ligand binding requirements for T cell activation and effects of signaling duration on induced gene expression.
- Detailed examination of individual T cell receptor (TCR)–peptide MHC (pMHC) binding events suggest that activation-inducing interactions are rare and require a single long dwell time or sequential, spatially correlated binding events.
- A rate-limiting step in proximal T cell signaling has been identified in the slow modification of the LAT residue that recruits PLCy1, suggesting a means for controlling the probability of activation based on TCR–pMHC interaction times.

## Outstanding Questions

- How are different molecular events co-regulated during T cell activation? What can be learned from multimodal measurements?
- Are T cell fates and activities downstream of activation also governed by conserved molecular programs regulated in a probabilistic fashion?
- To what extent does stimulation strength tune T cell responses beyond conserved activation programs, and how is this mediated?
- What is the molecular switch that turns on T cell activation?
- How do co-stimulatory signals feed into the intracellular processes of T cell activation?

## Box 1 Optogenetics

Optogenetic approaches take advantage of naturally light-responsive proteins to create synthetic systems that can be controlled with light of specific wavelengths. Optogenetics have been used to interrogate the organization of signaling networks across many biological fields^98^. The past few years have seen a rapid uptake of this technology for manipulating T cell signaling. For example, optogenetic manipulation of T cell calcium signaling in a spatially controlled manner has recently been achieved using a light-controlled STIM-1 construct that aggregates in response to two-photon stimulation^99^. Optogenetic approaches have been particularly informative for addressing questions of how the kinetics of receptor-ligand binding impact T cell activation responses. Studies using optogenetic receptors in the Jurkat T cell line and stimulating with cell-free ligands have been used to test the relationship between receptor-ligand binding kinetics and T cell activation^25,26^. Other studies have introduced cellular antigen presenting systems opposite light-responsive CARs to examine the impact of signal frequency and duration^23,24^. By necessity, these systems use synthetic receptors, which may show differences from native TCR-pMHC interactions, and this needs to be considered when interpreting results. However, the development of these methods marks an important new era in the study of T cell stimulation strength making it possible to precisely manipulate binding patterns under culture conditions that are otherwise identical. As such, the use of optogenetic systems has the potential to precisely define what we mean by stimulation strength.

## Box 2 Single-cell technologies

The last decade has seen rapid growth in technologies that enable high-dimensional molecular measurements in individual cells. Commercialization of several platforms has dramatically improved accessibility, increasing use across many fields, including fundamental T cell immunology.

Single-cell RNA sequencing (scRNA-seq) enables genome-wide transcriptome quantification within individual cells^100^. Originally, cells were processed in separate tubes or in multi-well plates for scRNA-seq (e.g. refs^101,102^). The subsequent development of droplet-based methods greatly increased the number of cells that can be sequenced per sample^103,104^. Novel methods and protocol refinements to improve transcript detection or cell throughput continue to be developed, including those specifically designed for CTLs^105^. Multi-modal measurements that quantify different types of features within individual cells can relate single-cell transcriptomes to other types of molecular measurements^106^, including protein expression^107,108^ and epigenetic modifications (e.g. refs^109,110^). These methods open the door to answering questions about gene expression regulation at the individual cell level.

Advances in cytometry techniques allow profiling tens of dimensions in thousands or millions of cells.

- Mass cytometry fuses flow cytometry and mass spectrometry to make targeted multidimensional measurements in individual cells^111-113^. Metal-conjugated antibodies or oligonucleotides allow simultaneous profiling of up to 57 markers of different molecular features, including protein expression, post-translational signaling protein modifications^114,115^, metabolic intermediates^116^, and mRNA transcripts^117^. Barcoding different samples with unique sets of metal isotope tags allows pooled staining and minimizes technical confounding^115^. High dimensional surface staining can resolve fine-grained cellular subpopulations^114,118^, while intracellular staining can capture complex multi-nodal signaling events^61,114,115^. An early mass cytometry study demonstrated the utility of this method for monitoring T cell signaling by comparing activating and inhibitory signals in a tumor-specific CTL clone stimulated with varied ligand doses^119^, while recent work has examined the impact of ligand affinity on naïve T cell activation^61^ (see main text). Combining molecular modalities within mass cytometry experiments allows comprehensive profiling of each cell to decipher regulatory logic on a single-cell level.
- Spectral flow cytometry records fluorescence across the spectrum at higher resolution than in conventional flow cytometry^120^. This allows analytical deconvolution of signals from each fluorescent marker and expands the dimensionality to be on par with mass cytometry. To date, this new technology has primarily been used for high-dimensional cell surface marker phenotyping (e.g. ref^121^), with exciting possibilities for RNA-flow cytometry.

By measuring protein epitopes, these new cytometry methods can provide important tools for creating a holistic picture of how stimulation strength impacts T cell activation events.

## Box 3 Live Imaging

Live microscopy acquires spatiotemporal information, allowing a sequence of events to be followed and identifying transient states that might otherwise be missed. Temporal information about a process we observe is crucial for proper analysis of static measurements made by other approaches (Figure 2). As such, many advances in microscopy have focused on improving temporal resolution while maintaining as high a spatial resolution as possible without damaging the specimen. This has led many investigations to use total internal reflection fluorescence (TIRF) microscopy, where only fluorophores within ~100 nm of the coverslip are illuminated^122^, achieving high temporal resolution at the expense of 3D measurements. While this approach provides an excellent signal to noise ratio for experiments such as direct visualization of binding events^74,123^ or measurement of force exerted on the TCR^124,125^, the artificial stimulatory surface does not have the biophysical properties of an antigen presenting cell and thus may perturb the very process being investigated^126^. To remedy this, new imaging technologies including lattice-light sheet microscopy^127^ capture multi-color 4D super-resolution images at high speed of interactions between live antigen presenting cells and T cells^128^. The increased volume of data from these approaches creates both challenges and opportunities for new analysis methods including machine learning^129^.

One T cell activation event for which live imaging measurements have proven particularly useful is the calcium flux. Downstream of TCR activation, the PI(4,5)P2 hydrolysis product (IP3) triggers calcium release from the endoplasmic reticulum, opening plasma membrane calcium channels and resulting in a further influx of calcium^130^. The importance of single-cell measurements to separate the magnitude of the calcium flux from its periodicity was first shown in 1998, when studies using either uncaging of IP3 or a calcium clamp approach showed that some transcription factors are most responsive to oscillations in the calcium flux and others to the amplitude^131-133^. Subsequent methods allowing direct visualization of the calcium flux demonstrated that as stimulation strength decreases, a larger proportion of cells show oscillatory rather than sustained calcium fluxes^134,135^. Further imaging advances have recently enabled simultaneous measurement of calcium signaling and centrosome movement in effector CTLs, suggesting a shared mechanistic link between the generation of prolonged calcium fluxes and docking of the centrosome at the immune synapse^63^. Expansion of such simultaneous imaging measurements will be critical for understanding the coordinated program of T cell activation.

## Glossary

### Abbreviations

Activation event: A measurable molecular change downstream of TCR stimulation in an individual T cell that marks its commitment to an activation program.
Activation rate: The number of T cells undergoing activation events per unit time.
Altered peptide ligand (APL): An MHC-binding peptide in which individual amino acid residues of the cognate peptide are altered, changing the TCR-pMHC ligand interaction and hence the T cell stimulation strength.
Analog response: The response exists on a continuum.
Calcium flux: Elevation of intracellular free [Ca^2+^].
Chimeric Antigen Receptor (CAR): An artificial receptor designed to target a specific protein and induce signaling similar to that downstream of a TCR.
Cytotoxic T lymphocyte (CTL): An activated T cell, secreting cytolytic components that elicit target cell death. While the majority of these are CD8^+^ effector T cells, CD4^+^ CTL also exist.
Digital response: The response is discrete, either seen or not seen, with no intermediary.
Dwell time: The time a T cell contacts an APC, or TCR contacts pMHC.
Effector T cell: A differentiated T cell providing a functional response.
Flow cytometry: Measurement of fluorescence intensity of individual cells, usually labeled with fluorescently bound antibodies, constructs or dyes.
Immunological synapse: The specialized interface formed between immune cells and their partners upon antigen recognition.
Kinetic proofreading: A mechanism for increasing ligand discrimination wherein the addition of reversible biochemical steps that delay the onset of further signaling enhances reliance of the pathway on receptor-ligand dwell time.
LAT signalosome: A multiprotein complex of proteins recruited to phosphorylated LAT.
Naïve T cell: A T cell that has yet to encounter a TCR antigen that it recognizes in the periphery.
Optogenetics: The introduction of light sensitive proteins into a cell to manipulate cellular behavior, e.g. the LOV2 photosensor domain from *Avena sativa* phototropin 1, or the phytochrome B-PhyB interacting factor ligand-receptor pair from *Arabidopsis thaliana*.
pMHC: Complex of peptide and MHC molecule.
Probabilistic model: The opposite of a deterministic model; a mechanism whereby inputs affect the probability of an output being generated. For the rate-based model of stimulation strength impacting T cell activation, increasing the strength of stimulation increases the probability of signals surpassing a molecular threshold(s) within each individual cell. This changes the percentage of individual T cell-APC interactions that initiate activation per unit time, altering the population response.
Pseudotime: A statistically inferred trajectory in which cells are ordered (and spaced) by the similarity of their molecular characteristics; when applied to cells undergoing a dynamic process, a trajectory constructed using a snapshot of heterogeneous cells at one real time can be postulated to correspond to how a cell might progress through the process.
Stimulation Strength: The integrated amount of activation-inducing signal a T cell senses through its TCR and other receptors sensing co-stimulation and the immune microenvironment.
Supported planar lipid bilayer: An artificial lipid membrane bilayer supported on a planar surface.
